# Wearables in Wales: Entering clinical practice through the backdoor?^☆^

**DOI:** 10.1016/j.fhj.2024.100014

**Published:** 2024-02-28

**Authors:** Jack Barrington, Zaheer Yousef, Christian Peter Subbe

**Affiliations:** a Betsi Cadwaladr University Health Board, Glan Clwyd Hospital, Bodelwyddan, Rhyl, LL18 5UJ UK; b Cardiff and Vale University Health Board Cardiff, UK; c Betsi Cadwaladr University Health Board, Bangor University, Bangor, Gwynedd, UK

**Keywords:** Cardiology, Arrhythmia, Wearables, Smartwatch, Wales

## Abstract

The number of people using wearable technology such as smartwatches and fitness trackers is increasing. Many of these devices can alert the user to a potential arrhythmia such as atrial fibrillation. We aimed to assess potential changes to cardiology practice resulting from their use.

An online questionnaire consisting of 12 questions was created and distributed to all consultant cardiologists in Wales. 25 of 102 participants replied, with 92% of these using consumer wearable technology to diagnose atrial fibrillation either ‘often’ or ‘sometimes’. One in four cardiologists received new referrals relating to wearable technology at least weekly.

The results demonstrate that cardiologists across Wales are using data from wearable technology in the diagnosis and management of cardiac conditions in the absence of formal guidance. Standardisation of guidelines and pathways is needed to support patients and clinicians and avoid the introduction of wearables through the unregulated backdoor.

## Introduction

An increasing number of people are using wearable technology such as smartwatches and fitness trackers. Recent research suggests that in 2022 there were over one billion wearable devices connected to the internet globally,^1^ and in 2023, sales of smartwatches are expected to reach 184 million.^2^ These wearable devices offer the potential for real-time monitoring of physical parameters, and many devices can monitor heart rhythm and record a basic electrocardiogram. This provides clinicians with an expanding reservoir of health-related data and new possibilities for diagnosing, monitoring, and managing various medical conditions. We aimed to investigate the current use and potential impact of data from wearable devices by consultant cardiologists working in the National Health Service in Wales.

## Methods

An online questionnaire was produced on Microsoft Forms using published guidance.^3^ The survey was distributed to all consultant cardiologists in Wales via email by the Welsh Cardiovascular Society in November 2022. This was a total of 102 recipients. Reminders to encourage completion were posted on Twitter.

The online questionnaire consisted of 12 questions (Appendix 1) focussing on the current utilisation of smart devices in clinical practice, frequency of referrals based on data from smart devices, presence of local guidelines on wearables, and free-text questions regarding any problems or concerns with the use of consumer wearable technology in cardiology practice.

## Results

25 respondents completed the questionnaire over a 4-week period in November 2022. This included consultants from all six Welsh health boards that have acute hospitals. 80% of the respondents represented consultants practising for more than 5 years. No further demographic data was collected to maintain anonymity. Among the consultants that responded, all survey questions were answered with no missing data.

**Fig. 1:**
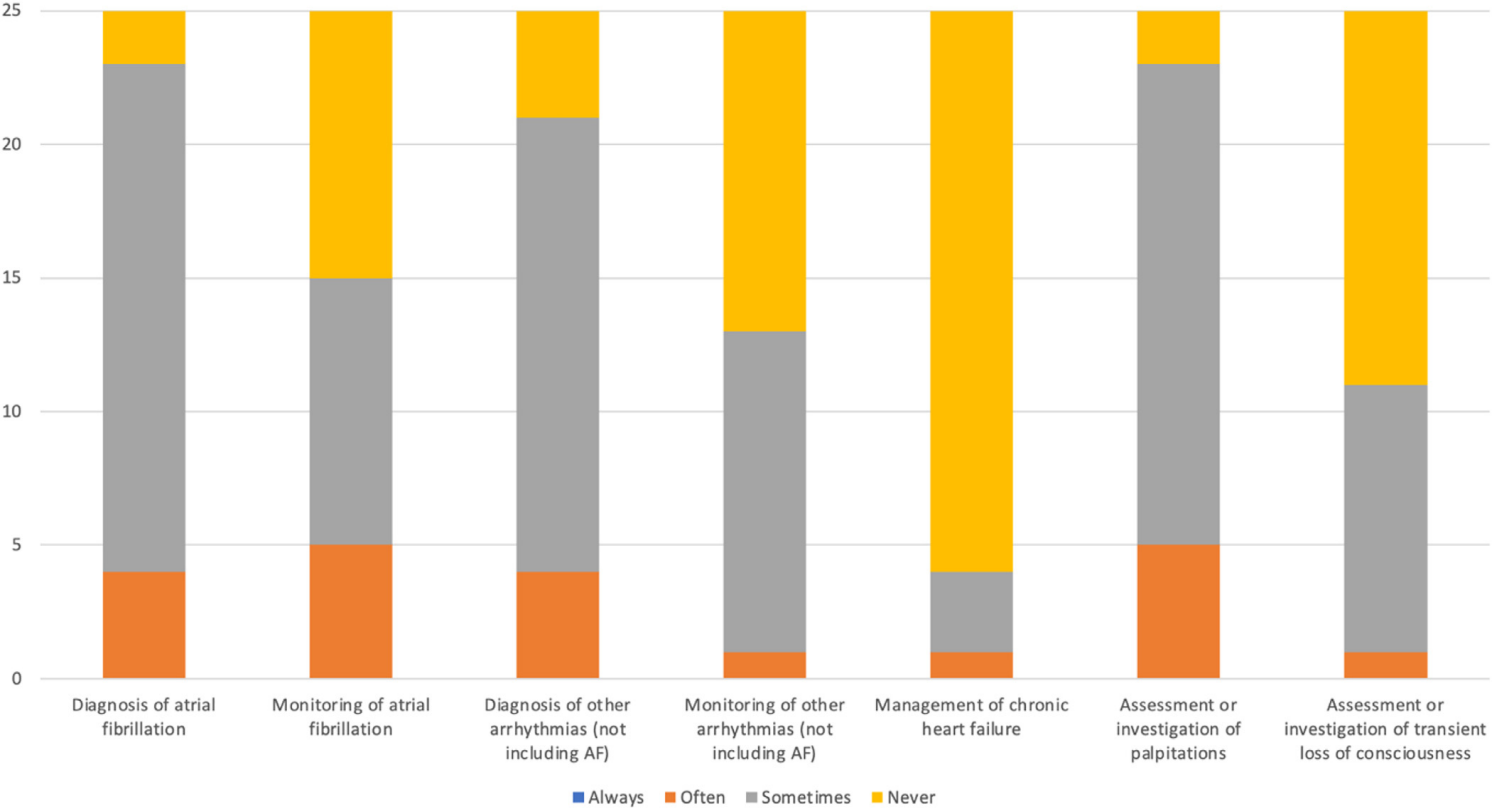
Which areas of your practice do you currently use data from consumer wearable technology?.

92% of respondents used consumer wearable technology to diagnose atrial fibrillation either ‘often’ or ‘sometimes’ (Fig. 1). 24% of respondents received at least one new referral relating to wearable technology at least weekly and 84% at least monthly.

Accessibility of the data from wearables is vital to its utility. Respondents rated accessibility on a ten-point scale with a mean of 5.2 (range 2–9, median 5). The majority of clinicians (44%) obtained wearable data through patients showing it on their wearable device, with 24% receiving data prior to the consultation and 20% receiving a printed copy from the patient during the consultation.

44% of respondents had diagnosed atrial fibrillation (AF) based on data from a patient's wearable device alone, without further investigations. 91% of these then went on to prescribe anticoagulation or change medications without any further investigations.

None of the respondents reported the existence of local guidelines regarding the diagnosis of AF based on data from consumer wearable technology. On a 10-point scale assessing the respondents' support for the incorporation of wearables into NICE guidelines, this was answered with a mean of 6.2 (range 0–10, median 7).

Contributors were asked whether they had ‘…encountered any problems with the use of consumer wearable technology in your clinical practice?’. Only five of the 25 participants denied problems. Consultants raised concerns about poor data quality for some products, but several praised the data quality from the market-leading provider.

When questioned on if they had ‘…concerns about the use of consumer wearable technology in clinical practice,’ participants expressed several different concerns. These included worries about elevating anxiety levels in patients and monitoring asymptomatic patients who would previously not have been monitored. A further point of concern was the medicolegal implications arising from the use of data that lacks validation for screening purposes.

One respondent shared an event of concern involving a patient with cardiac syncope secondary to a sinus arrest. The patient was simultaneously monitored by a Holter monitor, which recorded the event, and a wearable device, which failed to detect the event due to a lower sampling frequency. The brand of the wearable was not reported.

## Discussion

The results of our survey demonstrate that cardiologists across Wales are already using wearable technology in the diagnosis and management of cardiac conditions, with the majority receiving regular referrals based on data from wearables. This contrasts starkly with the reported absence of local guidelines.

One of the main areas of practice that our respondents utilise wearable devices is in the diagnosis of AF. Despite the prevalence of consumer wearable devices, the most recent NICE guidelines do not support their use by healthcare professionals for diagnosing AF.^4^ However, current European Society of Cardiology guidance states that a definitive diagnosis of AF can be made by a single-lead ECG tracing of ≥30 s from a wearable device.^5^ This discrepancy in guidance may explain how none of our respondents had local guidelines in place. Interestingly, there is a significant range of opinions on the incorporation of AF diagnosis from wearables into NICE guidance, and further research may be needed to explore the reasons for this.

The wEHRAbles 2 study^6^ assessed the current perspectives on wearable device heart rhythm analysis of 539 cardiologists worldwide: 83% stated they would diagnose AF based on a single-lead ECG tracing from a wearable device, with 73% of these initiating oral anticoagulation without further investigations. This is significantly higher than our data from Wales, with only 44% of respondents having made a diagnosis of AF based on wearable data alone. Whilst this is likely multifactorial, a documented limitation of the wEHRAbles 2 study was that most of their participants were electrophysiologists and therefore their results may not be generalisable to all physicians.^6^ Similar responses were seen between the two surveys for how clinicians obtain information from wearable devices, with most respondents in the wEHRAbles 2 study (63%) also being shown recordings on devices by patients during the consultation.

Alongside heart rhythm analysis, many wearable devices are also capable of monitoring a patient's vital signs. However, there is limited evidence on the use of this data in acute care, and further research is needed to determine the use of this data.^7^

Our survey raised concerns, including the potential for overdiagnosis and missed arrhythmias compared to traditional Holter monitors. Testing of wearables at scale and over longer periods of follow-up is needed to validate the data from wearables in healthcare.^8^

Our study has limitations. Using the Welsh Cardiovascular Society to distribute our questionnaire, alongside promoting the questionnaire on Twitter, we aimed to reach most consultant cardiologists in Wales. Despite this, we had a return rate of 25%, which introduces the potential for selection bias. Additionally, participation bias is a limitation, with responders of the survey potentially being biased towards early adopters and those using electronic media such as email and Twitter. Nevertheless, we hope that our work will stimulate discussion around this important and rapidly developing area of practice and pave the way forward for new technology to be integrated into clinical practice in a safe and standardised way.

## Conclusion

The widespread use of data from wearables by healthcare professionals in our survey may stimulate discussion around the introduction of formal guidelines. As the speed of development of new technology continues to increase, guidelines need to adapt at a similar pace to ensure standardised patient care.

## Figures and Tables

**Table:** 

	Question	Answer options
1	Which areas of your practice do you currently use data from consumer wearable technology? a) Diagnosis of atrial fibrillation b) Monitoring of atrial fibrillation c) Diagnosis of other arrhythmias (not including AF) d) Monitoring of other arrhythmias (not including AF) e) Management of chronic heart failure f) Assessment or investigation of palpitations g) Assessment or investigation of transient loss of consciousness	Never Sometimes Often Always
2	How often do you receive referrals based on data from consumer wearable technology?	Daily Weekly Monthly More than monthly Yearly Never
3	How accessible do you find patient data on their wearable device?	0–10 scale 0 = Not at all accessible 10 = Extremely accessible
4	How do you obtain data from patient's consumer wearable technology most frequently?	Patient brings printed copy to consultation. Patient shows data on device. Patient sends data prior to consultation. Not applicable. Other
5	Have you ever made a diagnosis of atrial fibrillation based on data from a patient's wearable device, without further investigations?	Yes No
6	If yes, did this lead to anticoagulation or any changes in medication without further investigations?	Yes No Not applicable
7	Do you have local guidelines regarding the diagnosis of AF based on the data from consumer wearable technology?	Yes No Don't Know
8	How much would you support the incorporation of formal guidance related to the diagnosis of AF based on data from consumer wearable technology into NICE guidelines?	0–10 scale 0 = Not at all likely 10 = Extremely likely
9	Have you encountered any problems with the use of consumer wearable technology in your clinical practice?	Free text answer
10	Do you have any concerns with the use of consumer wearable technology in your clinical practice?	Free text answer
11	Please select the statement that is most applicable to your current job role.	Consultant practising for MORE than 5 years. Consultant practising for LESS than 5 years. Specialty doctor practising for MORE than 5 years. Specialty doctor practising for LESS than 5 years. Cardiology registrar. Advanced Nurse Practitioner.
12	Which health board are you primarily based?	Aneurin Bevan University Health Board Betsi Cadwaladr University Health Board Cardiff and Vale University Health Board Cwm Taf Morgannwg University Health Board Hywel Dda University Health Board Powys Teaching Health Board Swansea Bay University Health Board I do not currently work in Wales
